# Circulating Hsp70 Reflects Tumor Burden and Stage-Dependent Disease Progression Across Multiple Solid Tumor Entities

**DOI:** 10.3390/cancers18091403

**Published:** 2026-04-28

**Authors:** Dominik Lobinger, Sophie Seier, Johanna L. Wolf, Nicholas Taylor, Karen Ainslie, Hannah Zanth, Ali Bashiri Dezfouli, Erika Roberts, Alan Graham Pockley, Hannah Herf, Luis Messner, Alexia Xanthopoulos, Christiane Guder, Merten Kliebisch, Gabriele Multhoff

**Affiliations:** 1 Department for Thoracic Surgery, Munich Clinic Bogenhausen, Academic Teaching Hospital Technische Universität München (TUM), 81925 Munich, Germany; 2 Central Institute for Translational Cancer Research of the Technische Universität München (TranslaTUM), TUM School of Medicine, TUM Universitätsklinikum, 81675 Munich, Germanynicholas.taylor@tum.de (N.T.); gabriele.multhoff@tum.de (G.M.); 3 Department of Otorhinolaryngology, Head and Neck Surgery, TUM School of Medicine and Health, TUM Universitätsklinikum, 81675 Munich, Germany; 4 Multimmune GmbH, 81675 Munich, Germany; graham.pockley@multimmune.com

**Keywords:** eHsp70, liquid biopsy, circulating biomarkers, tumor burden, cancer progression, extracellular vesicles, ROC analysis

## Abstract

**Background:** Liquid biopsy-based biomarkers provide valuable insights into tumor biology, dynamics, burden, relapse prediction and therapeutic responsiveness. The stress-inducible heat shock protein 70 (Hsp70), which is frequently overexpressed in highly aggressive solid tumors and is presented on the cell membrane of tumors but not normal cells, is found in the circulation either as a free protein originating from dying cells or in the context of extracellular vesicles (EVs) that are actively released by viable tumor cells. This study demonstrates the potential value of circulating Hsp70 (eHsp70) levels across multiple solid tumor entities as an entity- and stage-dependent diagnostic biomarker reflecting tumor burden and disease stage. **Methods:** Circulating eHsp70 levels, as determined using the Hsp70-exo ELISA which detects free and EV-associated Hsp70, in plasma samples collected from patients with different tumor entities (*n* = 389) prior to the initiation of any oncological therapy and healthy controls (*n* = 108) between 2021 and 2025, were analyzed retrospectively. Tumor stages were categorized as early, locally advanced, or metastatic. The Kruskal–Wallis test was used for group comparisons and the Receiver Operating Characteristic (ROC) curve was used to evaluate the diagnostic performance of eHsp70 levels. DeLong’s test was used to calculate differences between AUC values. **Results:** In tumor patients (*n* = 389), circulating eHsp70 levels were significantly higher than those in healthy controls (*n* = 108) (Kruskal–Wallis, *p* < 0.001). eHsp70 levels progressively increased from early-stage to locally advanced and metastatic disease in a stage-dependent manner. Although ROC analysis demonstrated the limited discriminatory performance of eHsp70 levels in early-stage disease (AUC 0.569), increased discrimination was apparent in locally advanced disease (AUC 0.751), metastatic tumors (AUC 0.784) and combined advanced tumor diseases (AUC 0.765; significant by DeLong’s Test comparing early-stage to locally advanced and metastatic tumors), irrespective of the tumor entity with the highest AUC values in metastatic breast cancer (AUC 0.872), sarcoma (AUC 0.861) and non-small cell lung cancer (NSCLC) (AUC 0.835). Apart from minor entity-specific differences, the correlation of eHsp70 levels with the tumor stage remained consistent across all measured tumor entities. **Conclusions:** Circulating eHsp70 levels are markedly elevated in patients with highly malignant solid tumors and show a consistent, stage-dependent increase across multiple tumor types. These findings suggest that circulating eHsp70, as an indicator of tumor-associated cellular stress and overall tumor burden, represents a valuable biomarker for assessing disease stage, monitoring disease progression, and evaluating therapeutic responses.

## Simple Summary

Reliable and minimally invasive biomarkers are urgently needed to improve cancer diagnosis and disease monitoring. Blood-based tests, known as liquid biopsies, offer the possibility to assess tumor burden without repeated invasive tissue sampling. In this study, we analyzed the molecular chaperone heat shock protein 70 (Hsp70) in the blood of patients with different types of cancer. We found that circulating Hsp70 levels (eHsp70) are significantly higher in cancer patients than in healthy individuals and increase with advancing disease stage. Although eHsp70’s ability to detect cancer at an early stage may be limited, it reflects tumor burden and disease progression in various types of tumors. These findings suggest that circulating Hsp70 could serve as a broadly applicable biomarker to support clinical decision-making, particularly for monitoring advanced disease and treatment response. Integrating eHsp70 into existing liquid biopsy approaches may improve the assessment of tumor dynamics in clinical practice.

## 1. Introduction

Cancer remains a leading cause of death globally, with an upward trend in many cases [1]. The urgent push to develop innovative diagnostic and therapeutic strategies [2] depends heavily on identifying suitable biomarkers. These biomarkers are essential not only for early tumor detection, but also for predicting how patients will react to different therapies, for monitoring treatment response, and for evaluating changes in tumor burden over time [3,4].

In recent years, interest in liquid biopsy-derived biomarkers including circulating tumor cells (CTCs), circulating tumor DNA (ctDNA), and tumor-derived extracellular vesicles (EVs) has grown dramatically. Screening for liquid biomarkers not only functions as a tool for detecting malignant diseases, but also as a means of revealing biological characteristics such as specific mutations, tumor burden and, in the best case, therapy response [5].

A promising candidate as a molecular biomarker is the evolutionarily highly conserved heat shock protein 70 (Hsp70), the major stress-inducible member of the 70 kDa heat shock protein family (HSP70) [6]. In addition to its physiological function as a chaperone in protein folding and protein homeostasis in all nucleated cells, Hsp70 influences multiple signaling pathways and cell proliferation, especially in tumor cells [7]. Multiple external stressors including hyperthermia and ischemia can trigger a dramatic upregulation of Hsp70 expression [8]. Furthermore, it is known that tumor cells of various entities exhibit significantly increased Hsp70 expression under physiological conditions [9,10,11,12]. Unlike normal cells, the localization of Hsp70 in tumor cells is not restricted to the cytosol. Tumor cells also express Hsp70 on the cell membrane and actively release it in lipid microvesicles via a non-ER mediated pathway [13,14,15]. An increased membrane Hsp70 expression density on tumor cells, which has been associated with unfavorable tumor stages and a poor prognostic outcome for some tumors, is associated with elevated circulating eHsp70 levels and levels might therefore serve as a liquid biopsy-based biomarker for evaluating the aggressivity of tumors [16,17,18,19].

Immunologically, membrane-bound Hsp70 on tumor cells acts as a target for activated natural killer (NK) cells [20,21]. However, NK cells in patients with late-stage tumors are often immunosuppressed, which hinders them in controlling tumor growth [22,23]. A clinical phase II study demonstrated enhanced killing of membrane-Hsp70-positive tumor cells and beneficial clinical effects in patients with late-stage NSCLC after ex vivo stimulation of immunosuppressed, autologous (patient-derived) NK cells with the Hsp70-peptide TKD and IL-2 [24]. To identify patients with membrane-Hsp70-positive tumors, circulating eHsp70 levels were determined using the Hsp70-exo sandwich enzyme-linked immunosorbent assay (ELISA) which measures microvesicle-bound Hsp70 actively released by living tumor cells, as well as free Hsp70 originating from dying tumor cells [25,26,27]. Previous data indicate that vesicular Hsp70 contributes the majority of the extracellular Hsp70 in the circulation of tumor patients [25].

In this retrospective analysis, we summarize eHsp70 levels in the circulation of 389 patients with different tumor entities in different stages before implementation of any kind of oncological therapy and a healthy control group to provide new insights into the role of eHsp70 values across multiple solid tumor types with a particular emphasis on entity- and stage-dependent differences and diagnostic performance.

## 2. Materials and Methods

### 2.1. Hsp70-exo Sandwich ELISA

The results of the eHsp70 levels measured using the Hsp70-exo sandwich ELISA in 389 patients between the years 2021 and 2025 were retrospectively analyzed and compared with those of 108 healthy donors. The procedure for the Hsp70-exo sandwich ELISA has been described previously and is again described in detail below. Circulating eHsp70 levels were measured at a single time point per patient prior to initiation of oncological therapy.

Plasma derived from EDTA anti-coagulated blood (S-Monovette, Sarstedt, Nümbrecht, Germany) was prepared by centrifugation at 1500× *g* for 15 min at room temperature, followed by aliquoting (300 µL) and storage of the plasma samples at −80 °C. The Hsp70-exo sandwich ELISA was performed in 96-well MaxiSorp Nunc-Immuno plates (Thermo, Rochester, NY, USA) using the cmHsp70.2 coating monoclonal antibody (mAb, 1 µg/mL) and the biotinylated cmHsp70.1 mAb (200 ng/mL; multimmune GmbH, Munich, Germany) in horseradish peroxidase (HRP)-Protector™ (Candor Bioscience GmbH, Wangen i. Allgäu, Germany) as the detection agent [19]. Briefly, plasma samples (100 µL) and a recombinant Hsp70 protein standard (1–100 ng/mL) were diluted in StabilZyme Select (1:5; Diarect GmbH, Freiburg i. Breisgau, Germany). After a 30 min incubation period at room temperature and washing, plates were incubated with Streptavidin (57 ng/mL, 30 min; Senova GmbH, Weimar, Germany) in HRP-Protector™ (Candor Bioscience GmbH, Wangern i. Allgäu, Germany). Colorimetric analysis was performed after adding the substrate reagent (100 µL, 15 min; BioFX TMB Super Sensitive One Component HRP Microwell Substrate, Surmodics, Inc., Eden Prairie, MN, USA). After quenching the reaction by adding 2 N H_2_SO_4_ (50 µL), the absorbance was read at 450 nm using a VICTOR X4 Multilabel Plate Reader (PerkinElmer, Waltham, MA, USA) and this was corrected using a reference absorbance at 570 nm [11,25].

### 2.2. Statistics

Statistical analyses were performed using the statistical software R (R Studio version 2025.09.2). Differences in absolute circulating eHsp70 levels were analyzed using Kruskal–Wallis testing. Discriminative performance of eHsp70 was analyzed using Receiver Operating Characteristic (ROC) curve analysis. Subsequently, the area under the ROC curve (AUC) and the corresponding 95% confidence intervals were calculated to quantify the ability to distinguish between malignant cases and healthy controls. Differences among AUC values were estimated using DeLong’s test. Results were visualized using forest plots summarizing AUC values and confidence intervals. *p*-values < 0.05 were considered statistically significant. Sample sizes used for ROC analyses are indicated in the respective figure legends. No correction for multiple comparisons was applied due to the exploratory nature of subgroup analyses.

### 2.3. Statement of Ethics

The analyzed plasma samples originated from previously approved sub-studies, each with independent ethical approval from the local ethics committees of the TUM School of Medicine and Health and the Charité University Hospital, Berlin. All sub-studies were conducted in accordance with the Declaration of Helsinki and written informed consent was obtained from all patients and healthy donors prior to inclusion. The present combined retrospective analysis was performed in accordance with the approvals of the respective local ethics committees, and no additional ethical approval was required.

## 3. Results

### 3.1. Analyzed Cohort

Circulating eHsp70 levels were measured in the plasma of a total of 389 patients with different malignancies before implementation of any kind of oncological therapy and 108 healthy donors. The cohort studied comprised 178 patients with non-small-cell lung cancer (NSCLC), 85 with head and neck cancer, 31 with prostate cancer, 26 with bladder carcinoma, 14 with pancreatic cancer, 9 with melanoma, 8 with mammary carcinoma, 8 with esophageal cancer, 8 with sarcoma, 8 with colorectal carcinoma, 6 with testicular cancer and 8 other individual cases (including thyroid cancer, renal cell carcinoma, urothelial carcinoma of the of the renal pelvis, thymus carcinoma, squamous cell carcinoma of the skin, multiple myeloma, and Merkel cell carcinoma). Tumor stages were harmonized across tumor entities using a simplified classification based on TNM criteria. In summary, 47 patients were diagnosed with early-stage disease (involving T1/T2, N0, M0 following the current TNM classification), 192 with locally advanced disease (T1/2, N+, M0 or T > 2, N0/N+, M0) and 139 with metastatic disease (M+). Table 1 summarizes the patient characteristics, tumor entities and the extent of the tumor disease classified as early-stage, locally advanced and metastatic. Cases categorized as “other” were excluded from entity-specific analyses due to heterogeneity, but were included in the overall cohort analysis. Multiple myeloma cases were not included in entity-specific analyses as the study focused on solid tumors.

### 3.2. Stage-Dependent Discriminative Performance of eHsp70 Levels

**Figure 1:**
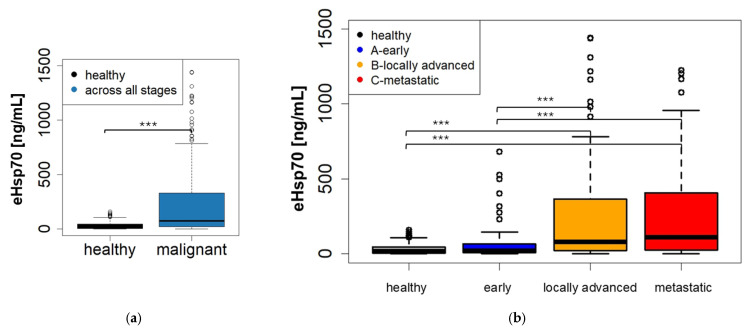
Circulating eHsp70 levels in patients with malignant disease and healthy donors. (**a**) Circulating eHsp70 concentrations measured by Hsp70-exo ELISA in plasma samples from patients with malignant disease (*n* = 389) compared to healthy donors (*n* = 108). Tumor patients showed significantly elevated circulating Hsp70 levels in comparison with healthy controls (Kruskal–Wallis test, *** = *p* < 0.001). (**b**) Stage-dependent distribution of circulating eHsp70 levels across all tumor entities. Patients were subdivided into early-stage (*n* = 47), locally advanced (*n* = 192) and metastatic disease (*n* = 139). A progressive increase in circulating eHsp70 concentrations was observed from early-stage tumors to locally advanced and metastatic disease. Statistical significance was assessed using Kruskal–Wallis testing.

Across all tumor entities, circulating eHsp70 levels were significantly higher in tumor patients (mean 249.37, median 72.8 ng/mL) compared to the healthy control cohort (mean 35.06, median 16.44 ng/mL, Kruskal–Wallis, *p* < 0.001), as shown in the boxplot in Figure 1a. The cohort of patients with various malignancies, including NSCLC, head and neck cancer, prostate cancer, bladder carcinoma, pancreatic cancer, melanoma, mammary carcinoma, esophageal cancer, sarcoma, colorectal carcinoma, and testicular cancer, showed a wider distribution in the eHsp70 levels, with some outliers exhibiting significantly elevated values.

A categorization of all tumor patients with different entities into early-stage, locally advanced and metastatic disease revealed a stage-dependent correlation of eHsp70 levels with a gradual increase from early-stage (mean 87.54, median 22.87 ng/mL) to locally advanced (236.86, median 80.4 ng/mL) and metastatic disease (mean 291.80, median 110.15 ng/mL) (Figure 1b). Significant differences in eHsp70 concentrations were observed between the healthy control group and between early-stage tumors compared to locally advanced and metastatic tumors (Kruskal-Wallis test, *p* < 0.001).

A Receiver Operating Characteristic (ROC) analysis determined the capacity of measured eHsp70 levels to discriminate between healthy donors and patients with malignant diseases.

**Figure 2:**
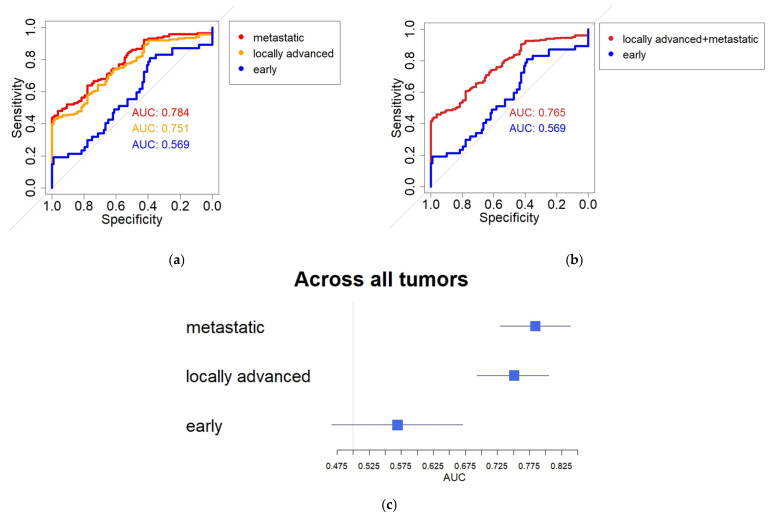
Stage-dependent diagnostic performance of circulating eHsp70. (**a**) Receiver Operating Characteristic (ROC) curves illustrating the discriminatory performance of circulating eHsp70 for early-stage, locally advanced and metastatic tumors. Optimal cut-off values were determined using the closest-to-top-left method and are reported in the Results Section. (**b**) ROC analysis comparing early-stage disease with combined advanced disease (locally advanced and metastatic tumors). Advanced disease demonstrated significantly higher discriminatory performance. (**c**) Forest plot summarizing the area under the curve (AUC) values and corresponding confidence intervals for different tumor stages, highlighting the stage-dependent diagnostic performance of circulating eHsp70. DeLong’s tests confirmed significant differences between early-stage disease and both locally advanced and metastatic tumors.

In line with the previous data, the discriminatory power of circulating eHsp70 levels increased with more advanced tumor stages. In the early stages of disease, the distinction between patients and healthy control subjects was limited (AUC 0.569), whereas discriminatory performance for locally advanced (AUC 0.751) and metastatic diseases (AUC 0.784), as well as locally advanced and metastatic diseases combined (AUC 0.765), was stronger (Figure 2a,b and Supplementary Figure S1).

DeLong’s test confirmed the described trend, whereby the AUC for early-stage disease was significantly lower than that of locally advanced disease (*p* = 0.0023), metastatic disease (*p* = 0.0003) and the two combined (locally advanced and metastatic disease, *p* = 0.0007). In this cohort of samples, no significant differences were found between locally advanced and metastatic diseases (*p* = 0.3957).

The forest plot shown in Figure 2c clearly demonstrates the stage-dependent discriminatory power of eHsp70 levels in distinguishing between healthy donors and patients with malignant diseases. In addition to the significantly higher AUC values described in Figure 2a,b, the narrower 95% confidence intervals in the metastatic (0.7292–0.8394) and locally advanced stages (0.6963–0.8052) are also notably different when compared to early-stage disease (0.4668–0.6711). These findings should be interpreted with caution due to the limited subgroup sizes. Using the closest-to-top-left approach, optimal thresholds for circulating eHsp70 were 8.91 ng/mL for early-stage disease (sensitivity 0.77, specificity 0.41), 40.31 ng/mL for locally advanced disease (sensitivity 0.64, specificity 0.71), and 43.74 ng/mL for metastatic disease (sensitivity 0.67, specificity 0.74). 

### 3.3. Heterogeneity of Discriminative Performance of eHsp70 Levels Across Different Tumor Entities

**Figure 3:**
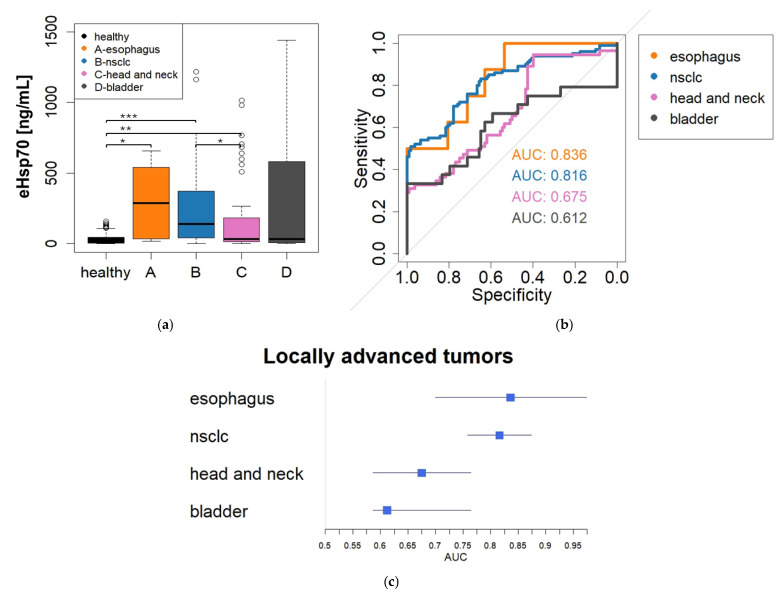
Heterogeneity of circulating eHsp70 performance across tumor entities. (**a**) Circulating eHsp70 concentrations in patients with locally advanced tumors from different entities (esophageal carcinoma—*n* = 8, NSCLC—n = 100, head and neck cancer—*n* = 55, bladder cancer—*n* = 24) compared with healthy donors. Statistical significance was assessed using Kruskal–Wallis testing (* = *p* < 0.05; ** = *p* < 0.01; *** = *p* < 0.001). (**b**) ROC curves illustrating the discriminatory performance of circulating eHsp70 for individual tumor entities in locally advanced disease. (**c**) Forest plot summarizing the area under the curve (AUC) values and corresponding confidence intervals of selected tumor entities, demonstrating significant differences between esophageal carcinoma and bladder cancer, NSCLC and bladder cancer, as well as NSCLC and head and neck cancer.

Patients with locally advanced tumors (esophageal carcinoma—mean 300.48 ng/mL, median 286.66 ng/mL; advanced NSCLC—mean 246.72 ng/mL, median 139.3 ng/mL; head and neck tumors—mean 169.02 ng/mL, median 30 ng/mL; bladder cancer—mean 329.5, median 31.26 ng/mL) showed elevated eHsp70 levels compared to healthy donors, with significantly different values between the control group and patients with esophageal tumors, those with NSCLC and those with head and neck tumors (Kruskal–Wallis, *p* < 0.05–0.001) (Figure 3a).

A closer look at the ROC analysis of patients with locally advanced tumors (Figure 3b) reveals histology-dependent heterogeneous AUC values: an AUC value of 0.836 for esophageal cancer, 0.816 for NSCLC, 0.675 for head and neck cancer and 0.612 for bladder cancer. Significant differences in AUC values were observed between selected tumor types, including esophageal cancer and bladder cancer (*p* = 0.036), NSCLC and bladder cancer (*p* = 0.017), as well as NSCLC and head and neck cancer (*p* = 0.0097). These results demonstrate the heterogeneous dynamics of circulating Hsp70 across different tumor types, while the general stage-dependent pattern is preserved. The forest plot in Figure 3c illustrates the different AUC values described and shows 95% confidence intervals with varying degrees of spread: esophagus AUC 0.6977–0.9735; NSCLC AUC 0.7583–0.8743; head and neck AUC 0.5864–0.7641; bladder cancer AUC 0.5864–0.7641.

**Figure 4:**
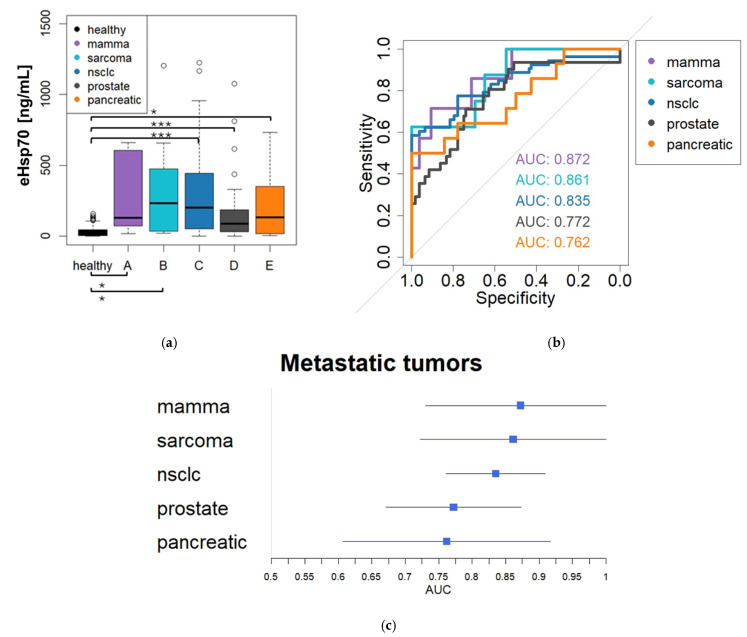
Diagnostic performance of circulating eHsp70 in metastatic tumor entities. (**a**) Circulating eHsp70 levels in patients with metastatic tumors (mammary carcinoma—*n* = 7, sarcoma—*n* = 8, NSCLC—*n* = 53, prostate cancer—*n* = 31, pancreatic cancer—*n* = 14) compared with healthy donors. Statistical significance was assessed using Kruskal–Wallis testing (* = *p* < 0.05; *** = *p* < 0.001). (**b**) ROC curves illustrating the discriminatory performance of circulating eHsp70 in metastatic tumor entities. (**c**) Comparison of AUC values across metastatic tumors with particularly high diagnostic performance observed in mammary carcinoma (AUC 0.872), sarcoma (AUC 0.861) and NSCLC (AUC 0.835).

The trend toward stage-dependent differentiation described above was also evident when examining various forms of metastatic diseases (mammary carcinoma, 309.02 ng/mL; sarcoma, 338.38 ng/mL; NSCLC, 413.23 ng/mL; prostate cancer, 201.41 ng/mL; pancreatic cancer, 246.98 ng/mL) with significant differences observed in all subgroups compared to healthy donors (Kruskal–Wallis, *p* < 0.05–0.001). (Figure 4a) An examination of the ROC analysis for patients with metastatic disease (Figure 4b) reveals the following AUC values: mammary carcinoma AUC 0.872, sarcoma AUC 0.861, NSCLC AUC 0.835, prostate cancer AUC 0.772 and pancreatic cancer AUC 0.762. Figure 4c shows a forest plot analysis of the metastatic tumors described, including the 95% confidence intervals (mammary carcinoma AUC 0.7302–1; sarcoma AUC 0.7217–1; NSCLC AUC 0.7607–0.9094; prostate cancer AUC 0.6714–0.8728; pancreatic cancer AUC 0.6067–0.9171). Additional ROC analyses and entity-specific discriminatory performance is shown in Supplementary Figure S2.

## 4. Discussion

In recent years, liquid biopsy approaches such as CTCs, ctDNA and cfDNA have increasingly been recognized as valuable indicators of tumor burden and disease progression and dynamics. One of the major advantages of liquid biopsies is that they can be used repeatedly without the need for invasive tissue sampling, which carries potential risks of tumor cell spread or injury to surrounding structures [5]. This allows higher frequency sample collection and regular monitoring of response during ongoing therapies [28]. Despite encouraging developments, there remains a shortage of dependable biomarkers that can reliably forecast how aggressive a tumor will be.

Previous studies have shown that eHsp70 can be actively secreted via EVs or released during cell death processes, such as necrosis and apoptosis. The majority of circulating eHsp70 originates from vesicular Hsp70 [29,30]. The unique Hsp70-exo ELISA enables levels of both free and vesicular eHsp70 to be measured in the blood of patients with tumors with a high sensitivity and reliability [25]. The analysis of microvesicle-associated biomarkers provides a unique opportunity as the signals provided by this type of analysis are fundamentally different to those provided by CTCs, ctDNA and cfDNA: CTCs are released from tumors that are in more advanced stages (i.e., metastatic, in the main) and ct/cfDNA originates from dying/dead cells, whereas microvesicles/exosomes are released from viable cells. The analysis of exosomal Hsp70 can therefore provide better insight into earlier stages of cancer growth (where no cell death is occurring and minimal amounts of cfDNA or CTCs are present) and increases/decreases in viable tumor cell size and growth. These characteristics are critical for any test that is to be used for detecting the effect of therapeutic interventions on viable tumor mass and identifying early disease recurrence.

Our study provides a comprehensive overview of the discriminatory performance of circulating stress-inducible eHsp70 in a large cohort of 389 patients across different tumor entities and tumor stages. In line with previous studies, circulating eHsp70 levels were significantly higher in patients with various malignant tumor diseases compared to healthy controls [12,31]. In smaller patient cohorts, a trend toward elevated eHsp70 levels was detected in patients with more aggressive tumors and a poorer prognosis [12,20,32]. In this study, we could show a clear stage-dependent increase in eHsp70 levels from early to locally advanced and metastatic tumors across all entities in absolute concentrations. With regard to diagnostic performance, ROCs demonstrated discriminatory power, showing an increase in locally advanced and metastatic stages of disease.

The observed stage-dependent differences in the eHsp70 levels are biologically plausible given the role of eHsp70 as a cellular stress protein. Tumor cells are exposed to multiple stressors, including hypoxia, metabolic changes and immune-cell mediated cytotoxicity, all of which can contribute to an increased membrane expression and release of eHsp70 [33]. The data are also in line with previous studies that have shown elevated Hsp70 expression on the tumor cell membranes to be related to an increased release of eHsp70 in more aggressive tumor types [30].

In addition to having a lower tumor cell burden, tumors in early stages may be exposed to lower levels of hypoxic and metabolic stress and may therefore release smaller amounts of eHsp70. In contrast, locally advanced and metastatic tumors are typically associated with higher proliferation rates, metabolic stress and areas of hypoxia, all of which might increase the release of eHsp70 into the circulation [34,35].

This interpretation is further supported by our forest plot analysis which showed wider confidence intervals and lower AUC values in early tumor stages compared to more advanced cases. These results therefore suggest circulating eHsp70 levels to be a suitable approach for assessing tumor burden, tumor stage and tumor aggressiveness. With respect to the finding that vesicular eHsp70 levels predominantly originate from viable tumor cells and therefore reflect viable and non-viable tumor burden, repeated measurements of eHsp70 might also be valuable for monitoring disease progression or therapeutic responses.

Another finding of the present data is the heterogeneity of the diagnostic performance of circulating eHsp70 in different tumor entities. Although elevated eHsp70 levels were observed in all malignancies, the discriminatory power varied between tumor types, with certain entities showing significantly higher AUC values than others. For example, significant differences were found between locally advanced esophageal cancer and locally advanced bladder cancer, as well as between locally advanced NSCLC and locally advanced bladder cancer and locally advanced head and neck cancer. These observations suggest that the systemic release of eHsp70 depends not only on the tumor stage but also on certain tumor-specific biological characteristics. The observed heterogeneity may reflect differences in tumor biology, peritumoral microenvironmental stress conditions and membrane-associated Hsp70 expression between different tumor types. Previous studies have shown that membrane-bound Hsp70 is expressed in a variety of solid tumors, but with varying frequency and density depending on the tumor entity and its biological aggressiveness [36,37,38]. Tumors characterized by high metabolic activity, hypoxia and increased cell turnover may therefore release larger amounts of eHsp70 into the circulation, both through passive release from dying cells and through active secretion via extracellular vesicles.

An important finding from the data is that, despite the entity-specific differences in eHsp70 expression described above, the overall stage-dependent pattern remained constant across all tumor types, thereby supporting the hypothesis that circulating Hsp70 levels primarily reflect tumor burden rather than tumor origin. Future studies focusing on larger entity-specific cohorts and additional molecular biomarkers could further investigate the role of tumor biological differences on the expression of eHsp70.

This study has several limitations. Demographic and clinical data are not available for all patients, so further adjustment for possible confounders such as age and gender was not possible and therefore could not be included in subgroup analyses. Furthermore, the study design, with eHsp70 levels assessed at a single time point per patient, and with no longitudinal measurements during disease progression available, does not allow conclusions to be drawn about changes in eHsp70 concentrations during and after treatment, nor on their ability to predict therapeutic response.

Despite these limitations, the present study provides a comprehensive characterization of circulating eHsp70 in different tumor stages and entities in a large cohort of cancer patients. The consistent stage-dependent pattern of measured eHsp70 concentration shown here underscores its biological relevance as a marker for tumor burden, aggressiveness, stage and tumor-associated stress.

A goal for future studies with larger cohorts will be to evaluate the longitudinal dynamics of eHsp70 at different points in time during therapy and to assess its inclusion in multi-marker liquid biopsy panels, thereby investigating its potential role in monitoring treatment response and disease progression.

## 5. Conclusions

Circulating eHsp70 levels measured using the Hsp70-exo ELISA, which can detect both free Hsp70 and EV-derived Hsp70, were significantly elevated in patients with malignancies compared to healthy donors. Although entity-specific discrepancies in diagnostic performance were shown, a constant stage-dependent pattern was observed for all tumor types with eHsp70 levels increasing progressively from early-stage disease to locally advanced and metastatic tumors. These findings indicate that circulating eHsp70 predominantly represents tumor burden, aggressiveness, stage and tumor-associated cellular stress and may therefore serve as a promising biomarker for assessing disease progression and tumor aggressiveness.

## Figures and Tables

**Table 1:** Overview of the analyzed study cohort including healthy donors and patients with different tumor entities. Tumor patients were categorized according to tumor stage: early-stage, locally advanced or metastatic disease. Circulating eHsp70 levels were analyzed in plasma samples collected prior to initiation of any oncological therapy. The table provides an overview of the number of patients with each type of tumor and at each stage.

Tumor Entity	Total (n)	Early-Stage (n)	Locally Advanced (n)	Metastatic (n)	Other (n)
Healthy donors	108	–	–	–	–
NSCLC	178	25	100	53	–
Head and neck cancer	85	22	55	5	3
Prostate cancer	31	–	–	31	–
Bladder carcinoma	26	–	24	2	–
Pancreatic cancer	14	–	–	14	–
Melanoma	9	–	–	4	5
Mammary carcinoma	8	–	–	7	1
Esophageal cancer	8	–	8	–	–
Sarcoma	8	–	–	8	–
Colorectal carcinoma	8	–	–	8	–
Testicular cancer	6	–	–	6	–
Other entities	8	–	5	1	2
Total tumor patients	389	47	192	139	11

## Data Availability

The datasets generated and analyzed during the current study are available from the corresponding author upon reasonable request.

## Supplementary Materials

The following supporting information can be downloaded at https://www.mdpi.com/article/10.3390/cancers18091403/s1: Figure S1: Circulating eHsp70 concentrations in patients with early and locally advanced plus metastatic tumors combined in comparison to the control cohort. Comparison analysis following Kruskal–Wallis testing showing statistical differences in circulating eHsp70 levels between analyzed groups. Figure S2: Additional ROC analyses and entity-specific comparisons supporting the stage-dependent discriminatory performance of circulating Hsp70.
