# Supplementary figures and images for: Circulating Hsp70 Reflects Tumor Burden and Stage-Dependent Disease Progression Across Multiple Solid Tumor Entities
Source: Cancers (Basel). 2026 Apr 28;18(9):1403. doi: 10.3390/cancers18091403 (PMC13163040; doi:10.3390/cancers18091403)

**Figure S1**

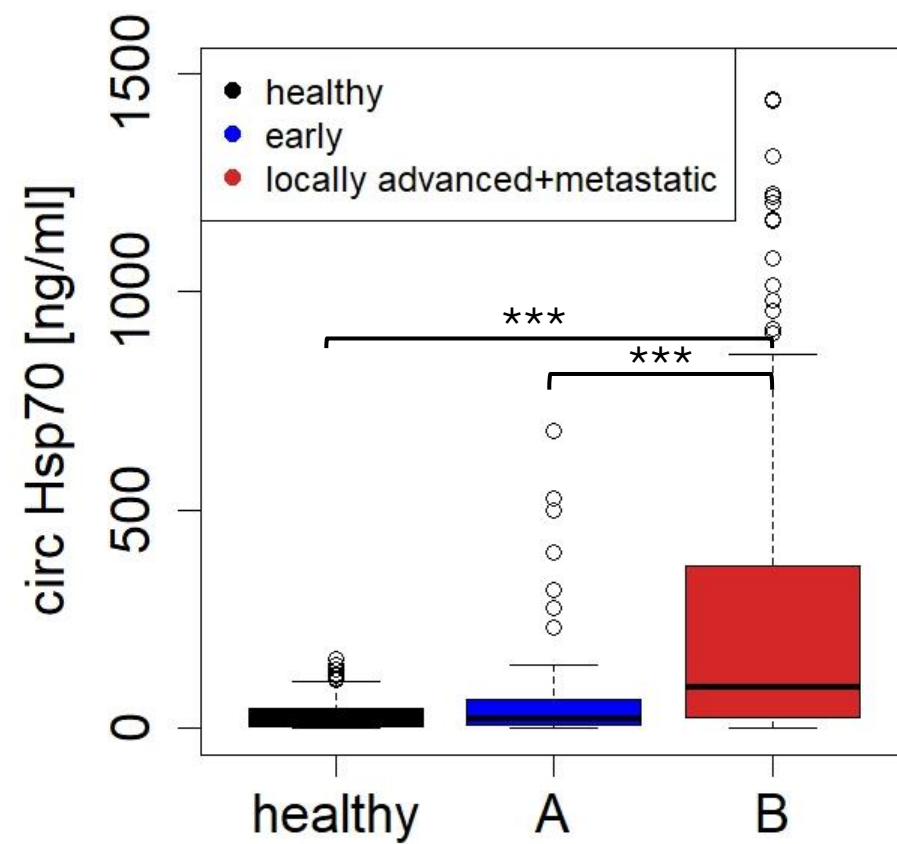

# Figure S2

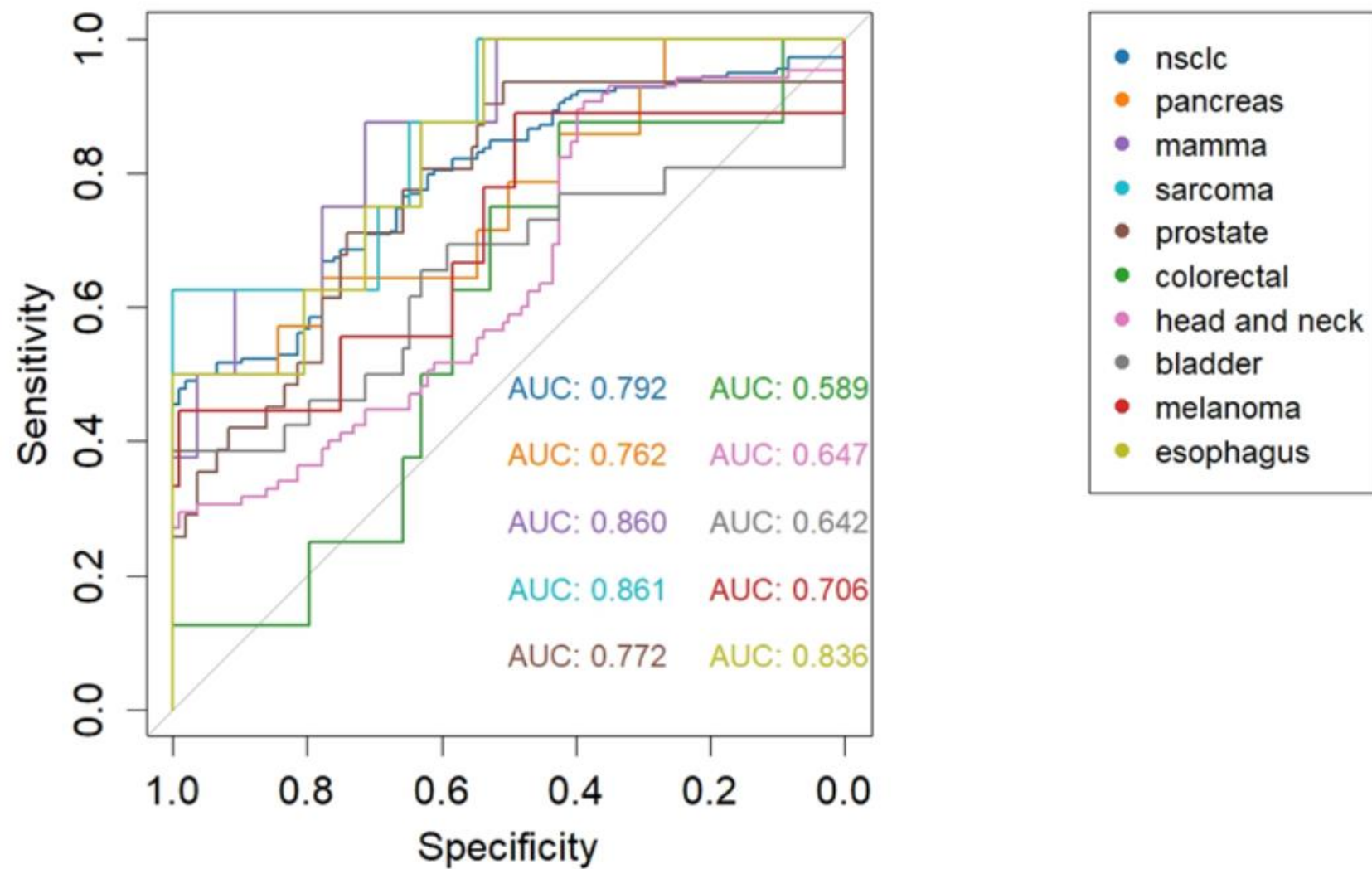

Supplement: Supplementary file 1 [file cancers-18-01403-s001.zip › cancers-4278385-supplementary.pdf]
